# Moral spillover in carbon offset judgments

**DOI:** 10.3389/fpsyg.2022.957252

**Published:** 2022-10-13

**Authors:** Patrik Sörqvist, Douglas MacCutcheon, Mattias Holmgren, Andreas Haga, Daniel Västfjäll

**Affiliations:** ^1^Department of Building Engineering, Energy Systems and Sustainability Science, University of Gävle, Gävle, Sweden; ^2^Department of Behavioural Sciences and Learning, Linköping University, Linköping, Sweden

**Keywords:** emotion, moral spillover, carbon offsets, moral motives, compensation estimates

## Abstract

Moral spillover occurs when a morally loaded behavior becomes associated with another source. In the current paper, we addressed whether the moral motive behind causing CO_2_ emissions spills over on to how much people think is needed to compensate for the emissions. Reforestation (planting trees) is a common carbon-offset technique. With this in mind, participants estimated the number of trees needed to compensate for the carbon emissions from vehicles that were traveling with various moral motives. Two experiments revealed that people think larger carbon offsets are needed to compensate for the emissions when the emissions are caused by traveling for immoral reasons, in comparison with when caused by traveling for moral reasons. Hence, moral motives influence people’s judgments of carbon-offset requirements even though these motives have no bearing on what is compensated for. Moreover, the effect was insensitive to individual differences in carbon literacy and gender and to the unit (kilograms or tons) in which the CO_2_ emissions were expressed to the participants. The findings stress the role of emotion in how people perceive carbon offsetting.

## Introduction

Moral spillover occurs when a morally loaded behavior becomes associated with another source. For example, moral spillover can occur when a person becomes more likely to cheat after recollecting another person’s immoral behavior (Mullen and Nadler, 2008). Another example of moral spillover can occur when a twin has done something morally questionable. People tend to intuitively associate the moral taint of a twin with that of the other twin (Uhlmann et al., 2012). Similarly, when confronted with human-induced harm to the environment, people can become more willing to engage in pro-environmental actions even when they are not themselves the ones responsible for the harm (Rees et al., 2015). In the current paper, we addressed moral spillover in a new setting. Specifically, we aimed to test whether the moral motive behind causing CO_2_ emissions (which is widely regarded as environmentally harmful and immoral) spills over on to how much people think is needed to compensate for the emissions.

People tend to seek balance in a moral account (Sachdeva et al., 2009; Ellemers et al., 2019). After doing something morally questionable, for instance, people are more willing to behave morally to gain back balance in the account (West and Zhong, 2015). Conversely, after doing something morally righteous (helping someone), there is a stronger tendency to engage in immoral actions (stealing), perhaps because people then feel morally licensed to do so (Merritt et al., 2010; Blanken et al., 2015). It does not seem to matter whether the moral and immoral behaviors come from the same or from different source domains. For example, if people are reminded of something they have done that is harmful to the environment, they tend to be more willing to behave in prosocial ways (Sachdeva et al., 2009).

The idea that good deeds can compensate for bad deeds (and vice versa) seems to underpin the way people process environmentally harmful and friendly behavior as well (Wolrath Söderberg and Wormbs, 2022). Taking the bicycle instead of the car to work for a few days a week, for example, can be seen as a way of compensating for the burden caused to the environment for taking the family on vacation by airplane (Kaklamanou et al., 2015; Hope et al., 2017; Sörqvist and Langeborg, 2019). Similarly, in the health domain, there is a tendency to think that unhealthy behaviors (drinking alcohol or smoking) can be neutralized by healthy behaviors (physical exercise), even though the harm caused to the body by the harmful behaviors and the gains from the healthy behaviors do not cancel each other out (Knäuper et al., 2003).

Behavior that causes carbon emissions, and their related carbon offsetting (actions taken to compensate for carbon emissions), are of particular interest to the current paper. People tend to perceive actions that are described as helpful to the climate—such as carbon offsetting—as inherently moral. One reason for this appears to be the desire to help create a caring society (Bain and Bongiorno, 2019). There are notable exceptions though. Some people think that carbon emissions have no effect on the climate (Gössling and Peeters, 2007). They are probably also morally indifferent to carbon emissions and to carbon offsetting. Moreover, the reasons for taking carbon offset actions vary. Some people do it for internal reasons, as part of their identity; others due to social pressure and external norms (Schwirplies and Ziegler, 2016). Carbon offsetting is thus often perceived as “the right thing to do,” either for internal or external reasons.

The moral dimension of carbon offsetting is also revealed by studies on the willingness to pay extra for carbon offsetting. Companies tend to offer consumers the opportunity to pay extra for carbon offsetting to compensate for the climate burden of using their services and purchasing their products as a business model for meeting environmental sustainability demands (Sapkota and White, 2020). Consumers’ willingness to pay for these schemes varies (Blasch and Farsi, 2014). Some consumers are willing to pay a premium for these carbon offset schemes (Hagmann et al., 2015), eco-centric consumers in particular (Mair, 2010). However, consumer’s willingness to accept carbon-offset premiums depend on extraneous factors as well. For example, those who feel normative social pressure to accept the offers are more likely to do so (Araghi et al., 2014). This suggests that preferences for carbon offsetting are not well-defined nor stable or pre-existing. Instead, preferences appear to be “constructed” based on available and salient information at the moment of making a judgment or decision (Slovic, 1995).

Compensation for environmental impacts is hence an issue that is associated with both moral values and moral emotions (Rees et al., 2015). Prior research in other domains, such as willingness to pay for environmental protection, has shown that moral emotions are a central input into the valuation process (Kahneman et al., 1998)—especially when people do not have a strong prior about the “value” of an action or the decision domain is abstract and unfamiliar (Slovic, 1995). Since emotion plays an important role to how people respond to information about climate change and environmental impact (Rees et al., 2015; Duan and Bombara, 2022), and carbon offsetting appears to be associated with moral emotions, we hypothesized that emotionally and morally salient information might influence estimates of carbon offset requirements, even when the emotional information is normatively irrelevant to the task. If compensation for environmental impact is cognitively processed as a moral issue, people may perceive a larger need for compensation when negative emotions become more salient, such as those in which environmentally harmful emissions come from actions that are morally questionable.

## Experiment 1

The first experiment was designed to test the novel hypothesis that people take the moral motive of an action into consideration when estimating how much carbon-offsetting is needed to compensate for the action’s emissions, even though the moral motive is normatively irrelevant. The hypothesis was that participants would report that more trees are needed to compensate for the emissions from a flight conducted with an immoral motive compared to the emissions from a flight conducted with a moral motive. Since the view of the relation between carbon emissions and moral stance varies considerably between people (Gössling and Peeters, 2007; Schwirplies and Ziegler, 2016), we expected the effect to be small but existent.

### Materials and method

#### Participants

A total of 500 participants (70.3% women, mean age = 33.64 years, *SD* = 11.17) took part in the experiment. All participants received a small monetary honorarium for their participation. Data was collected by an online questionnaire constructed by using the web-based questionnaire instrument Qualtrics. The questionnaire was distributed by the crowdsourcing platform *Prolific Academic* (Palan and Schitter, 2018). The inclusion criteria were having English as their first language, born and living in the U.K.

#### Materials, design, and procedure

A between participants design was used with one independent variable: reason for trip with two levels: moral and immoral.

At the start of the questionnaire, the participants were told that they would answer a question related to environmental impact. After responding to background information questions (i.e., age and gender), they were introduced to the compensation task. The response scale in the compensation task was inspired by Holmgren et al. (2018) wherein the participants were asked to state how many trees are needed to compensate for a set of carbon emissions. Carbon offsetting can come in many forms; one is reforestation (planting trees) which works as a carbon offsetting technique because trees bind carbon from the atmosphere. Planting trees as a carbon offsetting technique has been challenged on scientific and ethical grounds and may not be the best way to actually achieve carbon offsetting. Yet, it serves here simply as a tool to measure the dependent variable of the experiment. In the task, the participants were presented with the following statement: “*Please read this information carefully before proceeding: In this first part of the survey you will answers one question regarding environmental impact related to transportation. More specifically, you will be asked to estimate how many trees it would take to compensate for CO_2_-emissions as a result of a specific trip. Planted trees absorb CO_2_ for many years, and can therefore compensate for the negative environmental consequences of increasing CO_2_-emissions. Answer to the best of your abilities.*”

After reading the background information, participants were randomly assigned to one of two conditions. In the moral condition, they were presented with the compensation task stating: “*An aeroplane travels from Aden, Yemen to Paris, France with the purpose of rescuing child refugees from their war-stricken homeland and produces 1.2 tons CO_2_ emissions.”* In the immoral condition, the compensation task stated: “*An aeroplane travels from Paris, France to Aden, Yemen with the purpose of deporting child refugees back to their war-stricken homeland and produces 1.2 tons CO_2_ emissions.*” After the statement, the participants were presented with the question: “*How many trees are needed to compensate for this flight?*” with a scale ranging from 1 tree to 100 trees (endpoints labeled). They made their estimates on a slider scale, and the marker was set to the default value of “50 trees” prior to making the judgment. The participants adjusted the marker to align with their estimate. It took about 5 min to complete the survey.

### Results and discussion

**FIGURE 1:**
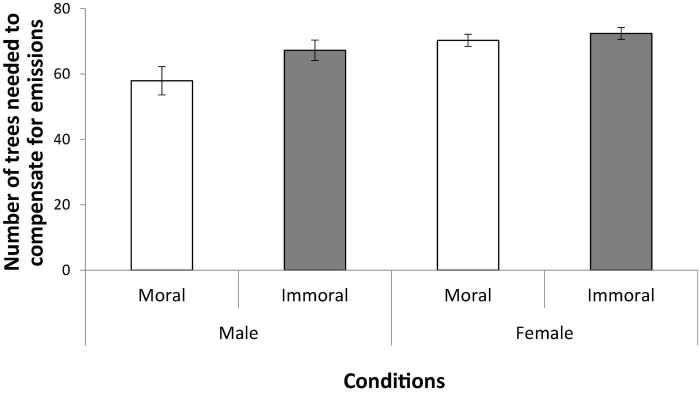
Mean estimates of how many trees needed to compensate for the emissions of an airplane trip made for either a moral or an immoral reason. Gender and moral reason main effects were significant, with no significant interactions. Error bars represent the standard error of the mean.

Since women are generally more concerned with environmental and climate change issues than men are (Mohai, 1992; Knez et al., 2013; Lee et al., 2013) and there are gender differences in moral reasoning and empathy (e.g., Löffler and Greitemeyer, 2021), data were separated according to gender in the analysis. As can be seen in Figure 1, the participants stated that more trees are needed to compensate for the carbon emissions from flights made with immoral motives in comparison with flights made with moral motives. There was also a difference between genders. These conclusions were supported by a 2 (Motive condition: moral vs. immoral) × 2 (Gender: male vs. female) analysis of variance. The analysis revealed a main effect of motive condition, *F*_(1, 494)_ = 4.68, *p* = 0.031, η*_p_*^2^ = 0.01, and a main effect of gender, *F*_(1, 494)_ = 11.02, *p* = 0.001, η*_p_*^2^ = 0.02, but no interaction between the two factors, *F*_(1, 494)_ = 1.88, *p* = 0.171, η*_p_*^2^ < 0.01.

The effect was very small in magnitude, but the results show that the moral motive behind environmentally harmful actions can influence people’s view of how much carbon offsetting is needed to compensate for the actions’ emissions. Moreover, people tend to take social and environment-related issues (information from two separate domains) into the mix even though the moral motives (rescuing vs. deporting children) have nothing *per se* to do with what is actually compensated for (emissions from the airplane).

As expected, female participants assigned a higher number of trees compared with men, in view of females being more concerned with the environment (Lee et al., 2013). However, the difference between the moral and immoral conditions had the same direction for both genders and the gender factor did not interact with the difference between the two experimental conditions.

## Experiment 2

The second experiment served three purposes. First, since the effect of moral motive on carbon offset reasoning is a novel finding, the follow-up experiment served the purpose of replicating this finding in order to reinforce reliability. This seemed particularly important in view of the small effect size. Second, we introduced a morally neutral condition to test whether the effect is driven by the immoral or by the moral motive. Third, the second experiment was designed to control for a number of factors. One factor shown previously to influence how people perceive carbon emission information is the so-called “unit effect” (Cadario et al., 2016). The measurement unit of the emissions (i.e., if expressed as kilograms or as tons) might bias estimates, causing higher estimates in conditions stating the emissions in kilograms than in conditions stating emissions in tons. Experiment 2 took this into consideration to test whether the moral bias observed in Experiment 1 is sensitive to such detail. Experiment 2 was also designed to control for some individual differences. Gender was again included in the analysis. Furthermore, the participants’ carbon literacy was measured (Sharp and Wheeler, 2013). A person’s carbon literacy corresponds to the persons’ general knowledge of true carbon emissions. This knowledge could influence how many trees people think are needed to compensate for emissions and it might influence how susceptible people are to the moral and immoral motives behind the things from which the emissions arise. Higher carbon literacy might make people less susceptible to such extraneous information. Because of this, we wanted to include carbon literacy in the analysis to test whether the moral bias remains even when carbon literacy is controlled for.

### Materials and methods

#### Participants

A total of 603 participants (67.7% women, mean age = 34.60 years, *SD* = 12.12) took part in the experiment. All participants received a small monetary honorarium for their participation.

#### Materials and procedure

Data was collected as in Experiment 1 with a few exceptions. Before being presented with tasks, participants were told that they would answer several questions related to environmental impact. After responding to background information questions (i.e., age and gender), they were introduced to the compensation task, stating: “*Please read this information carefully before proceeding: In this first part of the survey, you will answer questions regarding environmental impact related to different transportation means and distances. More specifically, you will be asked to estimate how many trees it would take to compensate for CO_2_-emissions as a result of different trips made by planes, buses and ships. Planted trees absorb CO_2_ for many years, and can therefore compensate for the negative environmental consequences of increasing CO_2_-emissions. Answer to the best of your abilities.*”

##### Compensation task

After reading the background information, participants were assigned to one of six conditions. In the moral and kilograms condition, they were presented with the compensation task, comprising of six different estimates. The first stated: “*An aeeroplane travels from Paris, France to Aden, Yemen with the purpose of delivering food to starving children and produces 1,200 kg CO_2_ emissions*,” the second stated “*A bus travels from Austin, Texas to Los Pobladores, Mexico with the purpose of building schools in a poor community and produces 1,000 kg CO_2_ emissions.*” The third stated: “*A ship travels from the coast of Baltimore, Maryland to the coast of Mogadishu, Somalia with the purpose of delivering medical supplies to hospitals in need of medicine and produces 2,100 kg CO_2_ emissions.*” The fourth stated: “*A bus travels from Brasilia, Brazil to Caracas, Venezuela with the purpose of delivering food to starving people and produces 1,100 kg CO_2_ emissions.*” The fifth stated “*An aeroplane travels from Toronto, Canada to Aleppo, Syria with the purpose of rebuilding hospitals which were destroyed in the war and produces 2,500 kg CO_2_ emissions.*” Finally, the sixth stated: “*A ship travels from the coast of Lisbon, Portugal to the coast of Bissau, Guinea-Bissau with the purpose of delivering medical aid to the population and produces 1 500 kg CO_2_ emissions.*” The immoral and kilograms condition had exact same estimates as the moral and kilograms condition but with immoral reasons stating: (1) *providing military weaponry for the ongoing war*; (2) *carrying crude oil*; (3) *delivering equipment for the construction of a new coal mine*; (4) *carrying palm oil for distribution; (5) disposing used electronics*; (6) *and relocating an isolated indigenous tribe due to planned road construction*. Note that the transportation means and emissions were the same order as in the moral and kilograms condition. The morally neutral and kilograms condition did not have stated reasons for the trips (e.g., *An airplane travels from Paris, France to Aden, Yemen and produces 1 200 kg CO_2_ emissions*). The three other conditions had the exact same types of estimates but stated the emissions in tons instead (e.g., 1,000 kg would be stated as 1 ton). Note that all six statements/scenarios were presented in all six conditions (and varied slightly in way of presentation as detailed above), so each participant made six estimates but only took part in one condition. After each of these statements, participants were presented with the question: “*How many trees are needed to compensate for this flight?*” (Note that the word “trip” was used instead of flight when the means of transportation was not by airplane) with a scale ranging from 1 tree to 100 trees (endpoints labeled). The participants made their estimates on a slider scale, and the marker was fixed at “1 tree” at the time of their judgment (see Supplementary Appendix for a visual representation of how the tasks were presented to the participants).

##### Carbon literacy questionnaire

After conducting the compensation task, participants were asked to answer two questionnaires measuring their carbon literacy. Both carbon literacy questionnaires were derived from Sharp and Wheeler (2013). The measurements used were the self-assessed knowledge and attitudes scale and objective carbon literacy measures (Sharp and Wheeler, 2013, p. 248, see Supplementary Appendix).

#### Design

A between participants design was used with two independent variables that were manipulated experimentally. The first was moral reason for trip with three levels (moral, neutral and immoral). The second variable was emission unit with two levels (kilograms or tons). Participant gender was also included in the analysis as a third independent variable. Five participants, who did not wish to state their gender, were removed from the analysis.

The participants were randomly assigned to one of six experimental conditions: moral and kilograms condition (*N* = 102), morally neutral and kilograms conditions (*N* = 99), immoral and kilograms condition (*N* = 101), moral and tons condition (*N* = 102), morally neutral and tons condition (*N* = 100), and the immoral and tons condition (*N* = 99). Each participant made 6 estimates (one for each scenario, as presented above) in the condition they were assigned to. That way, all scenarios were presented in all conditions. The scenario presentation order was randomized between participants. After the data were collected, the responses from the six estimates were collapsed to obtain a single, average estimate from each participant. As in Experiment 1, the participants were recruited by and responded to the questionnaire *via* the crowdsourcing platform *Prolific Academic*. The inclusion criteria were having English as their first language, born and living in the U.K., and each participant could only take part in one of the six different conditions. It took about 15 min to complete the survey.

### Results and discussion

As a check of response consistency, we first looked at the participants’ responses to the six individual scenarios. In the scenarios, the participants were asked to estimate how many trees were needed to compensate for a trip to Toronto (2,500 kg/2.5 tons CO_2_), to Baltimore (2,100 kg/2.1 tons CO_2_), to Lisbon (1,500 kg/1.5 tons CO_2_), to Paris (1 200 kg/1.2 tons CO_2_), to Brasilia (1,100 kg/1.1 tons CO_2_), and to Austin (1,000 kg/1.0 tons CO_2_). The mean estimates (when moral motive condition and emission unit condition were disregarded) were as follows for the various destinations: 66.27 trees (*SD* = 29.49) for Toronto, 61.70 trees (*SD* = 29.79) for Baltimore, 51.69 trees (*SD* = 29.09) for Lisbon, 50.36 trees (*SD* = 29.42) for Paris, 45.19 trees (*SD* = 29.23) for Austin, and 43.58 trees (*SD* = 28.71) for Brasilia. Higher emission magnitudes were thus systematically related to a larger estimate. The correlation between emission size and estimation size was strongly significant (*r* = 0.987), suggesting that participants adjusted their responses in a consistent manner in response to changes in emission size.

**FIGURE 2:**
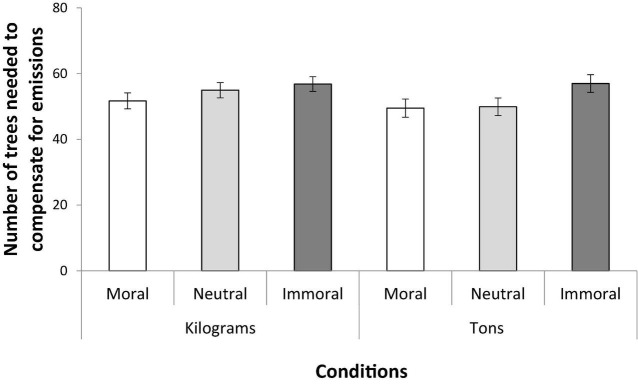
Mean estimates of how many trees needed to compensate for the emissions (stated in either kilograms or tons) from trips. Either the trips had a moral, neutral, or an immoral reason. Only the moral reason main effect was significant, and there were no significant interactions. Error bars represent the standard error of the mean.

As can be seen in Figure 2, there was a main effect of moral reason for the trips. This result was demonstrated by a 3 (Moral motive: moral vs. neutral vs. immoral) × 2 (Emission unit: kilograms vs. tons) × 2 (Gender: male vs. female) univariate analysis of variance. There was a main effect of moral motive, *F*_(2, 586)_ = 4.46, *p* = 0.012, η*_p_*^2^ = 0.02, but no main effect of emission unit, *F*_(1, 586)_ = 0.25, *p* = 0.621, η*_p_*^2^ < 0.01, nor an interaction between these two factors, *F*_(2, 586)_ = 0.19, *p* = 0.828, η*_p_*^2^ < 0.01. Moreover, there was no effect of gender, *F*_(1, 586)_ = 1.35, *p* = 0.245, η*_p_*^2^ < 0.01, nor an interaction between gender and moral motive, *F*_(2, 586)_ = 1.15, *p* = 0.317, η*_p_*^2^ < 0.01, or between gender and emission unit, *F*_(1, 586)_ = 0.97, *p* = 0.324, η*_p_*^2^ < 0.01. The analysis did not reveal a three way interaction, *F*_(2, 586)_ = 0.32, *p* = 0.726, η*_p_*^2^ < 0.01.

*Post hoc* independent samples *t*-tests showed a difference between the moral and immoral reason for the trip. Participants estimated that less trees were needed to compensate for the trip when it was made for moral reasons compared with when it was made for immoral reasons, *t*(402) = 2.49, *p* = 0.013, Cohen’s D = 0.25. There was a statistical tendency for a similar difference between the immoral and the neutral reason for the trip, *t*(402) = 1.81, *p* = 0.072, Cohen’s D = 0.18, that would be regarded as statistically significant on the notion of a one-tailed hypothesis. There was no difference between the moral and the neutral reason for the trips, *t*(401) = 0.71, *p* = 0.479, Cohen’s D = 0.07.

Furthermore, the data showed a significant negative relationship between mean estimates of trees (*M* = 53.13, *SD* = 25.47) and the participants’ level of objective carbon literacy (*M* = 7.14, *SD* = 2.63), *r*(590) = −0.13, *p* = 0.002, indicating that participants who had higher scores in carbon literacy reported fewer trees in general (six participants did not complete the objective carbon literacy measure and data are therefore missing from them in the analysis). The self-assessed knowledge and attitudes scale (*M* = 6.23, *SD* = 1.19) was unrelated to mean estimates of trees, *r*(596) = −0.01, *p* = 0.766. To control for the potential influence of carbon literacy on the difference between the moral motive conditions, we ran a 3 (Moral motive: moral vs. neutral vs. immoral) × 2 (Emission unit: kilograms vs. tons) × 2 (Gender: male vs. female) analysis of covariance (ANCOVA) with objective carbon literacy as the covariate. The addition of the covariate did not change any outcome. Most importantly, the main effect of moral motive for the trips was still significant, *F*_(2, 579)_ = 4.44, *p* = 0.012, η*_p_*^2^ = 0.02.

In sum, Experiment 2 replicated the novel finding that the moral motive behind actions can influence people’s view of how much carbon offsetting is needed to compensate for emissions, even though the moral motives have no bearing on what is compensated for. The effect is small but appears to be robust to the potential influence from individual differences in carbon literacy, factors related to gender and the unit by which the emissions are communicated to the person making the estimate.

## General discussion

We show that the moral valence of the motive behind environmentally costly trips can influence people’s view of how much carbon offsetting is needed to compensate for the emissions from those trips, even when the morality of a trip clearly has no bearing whatsoever on its total carbon emissions and subsequent offsetting requirements. The influence of morality remained when controlling for participants’ carbon literacy, indicating that being knowledgeable about the true impacts of things and behaviors did not dampen the effect of the moral cue on offsetting estimates; this in itself represents an interesting departure point for future research. This form of systematic “moral bias” on cognitive judgments has potential consequences for sustainable behavior and decision-making, giving the present study a high degree of contemporary relevance as both consumers and policymakers struggle to enact environmentally beneficial changes and meet carbon emission targets.

The results reported here add to the growing body of evidence suggesting that evaluations of the environmental impact of “green” items can be irrationally underestimated, implying cognitive interference due to this pro-environmental, and therefore inherently moral, association (MacCutcheon et al., 2020; Sörqvist et al., 2020). The present study goes one step further and shows that this calculation error can be incurred even when keeping the environmental impact of both comparison conditions constant, simply by manipulating the moral valence of associated information. In a broad sense, the phenomenon observed in the current series of studies can be seen as an instance where there is spillover from the moral reason behind an action to estimates of compensation requirements. If the action is immoral in one dimension (social), that appears to spill over to the action’s moral severity with regard to other dimensions (environmental). It’s interesting to note that causing CO_2_ emissions in itself is widely regarded as immoral because it is harmful to the environment/climate. This circumstance may be what makes estimates of carbon-offsetting requirements (i.e., estimates of what is required in order to compensate for something morally disgraceful) susceptible to the influence this moral bias. If the estimates would be about something that has nothing to do with morality, then it is likely that the estimates would be immune to such moral bias. For example, if people would be asked to estimate the value of art transported with flights made for various moral reasons, such estimates would probably be immune to any influence from the moral motives of the trips. Testing these boundary conditions for the moral spillover effect observed here could be another departure point for future research.

Taken together, the results from the two experiments suggest that the moral bias in carbon-offset judgments appears to be relatively insensitive to a number of factors. It seems insensitive to individual differences in carbon literacy and gender. Moreover, whether the information on carbon emissions is expressed as kilograms or as tons does not seem to modulate the moral bias. The only robust effect reported here was the one attributable to the moral valence of the reasons for the trips on participants’ estimates of carbon offset requirements. The findings thus stress the influence of emotion on cognitive operations. Greenwashing (i.e., misleading consumers about the environmental benefits of products) has proven to be an effective marketing strategy precisely because it induces exaggeratedly positive consumer sentiment associated with pro-environmental stimuli, triggering an “optimistic bias” whereby consumers overestimate the benefits of allegedly environmentally beneficial products (Delmas and Burbano, 2011), leading to vast but ill-gotten financial gains. This overestimation is likely due to the bypassing of systems for rational thinking through the triggering of emotion; to participants that are bombarded daily with affect-inducing media items about climate concerns, melting glaciers and mass extinctions, the emotion-inducing effect could be particularly pronounced. Similarly, the triggering of emotion could explain why participants seem to default to heuristics when presented with immoral stimuli rather than make the cognitively demanding calculations involved in estimating the offsetting requirements for the various trips in the study.

The results could also have some potential implications for consumer’s willingness to accept carbon-offsetting schemes. Voluntary carbon-offsetting is reinforced by social pressure (Araghi et al., 2014) and eco-centric attitudes (Mair, 2010). Before deciding to engage in a morally loaded behavior (such as paying a premium for carbon-offsets), people evaluate the costs in relation to potential gains of moral cleansing (cf. West and Zhong, 2015). If the gains are low and the costs are high, then the willingness to behave correspondingly should arguably decline. The results reported here indicate that willingness to accept carbon-offset premiums could depend, not only on the offsets’ environmental benefits, but also on other factors that are unrelated to the environment. Marketing social responsibility could, possibly, also increase consumers’ willingness to accept carbon-offset premiums. This is another avenue for future research.

Finally, a theoretically interesting avenue for future research would be to look at the accuracy of the participants’ estimates in the compensation task. The amount of carbon that a tree actually binds depends on many factors, such as the type of tree, its size and lifetime. It is therefore difficult to determine exactly how many trees are actually needed to compensate for a specific amount of carbon emissions. Therefore, future research could design a task that allows for an analysis of the participants’ accuracy in their compensation estimates and an analysis of whether morally loaded information increases or decreases estimation accuracy.

## Conclusion

The results reported here stress the role of emotion and morality in how people perceive carbon offsetting. Thus, the findings add to the body of evidence suggesting that emotions provoked by morally valenced stimuli can play an important part in climate change mitigation strategies and the promotion of pro-environmental action (Rees et al., 2015).

## Data availability statement

The raw data supporting the conclusions of this article will be made available by the authors, without undue reservation.

## Ethics statement

Ethical review and approval was not required for the study on human participants in accordance with the local legislation and institutional requirements. The patients/participants provided their written informed consent to participate in this study.

## Author contributions

PS wrote large parts of the manuscript and supervised design and analysis. DM generated the original idea, designed the experiments, and wrote parts of the manuscript. MH conducted data collection, designed the experiments, analyzed the data, and wrote parts of the manuscript. AH wrote parts of the manuscript. DV wrote parts of the manuscript and made significant comments on the manuscript. All authors contributed to the article and approved the submitted version.
