# Supplementary material for: Moral spillover in carbon offset judgments
Source: Front Psychol. 2022 Oct 13;13:957252. doi: 10.3389/fpsyg.2022.957252 (PMC9608638; doi:10.3389/fpsyg.2022.957252)
Supplement: Supplementary file 1 [file Presentation_1.pdf]

Appendix

The slider response scale used in Experiment 2 is depicted below together with corresponding questions/scenarios. Note that the order of questions/scenarios was randomized between participants. The slider task in Experiment 1 was identical with the appurtenant text (see Method section) but with an anchor in the middle of the scale.

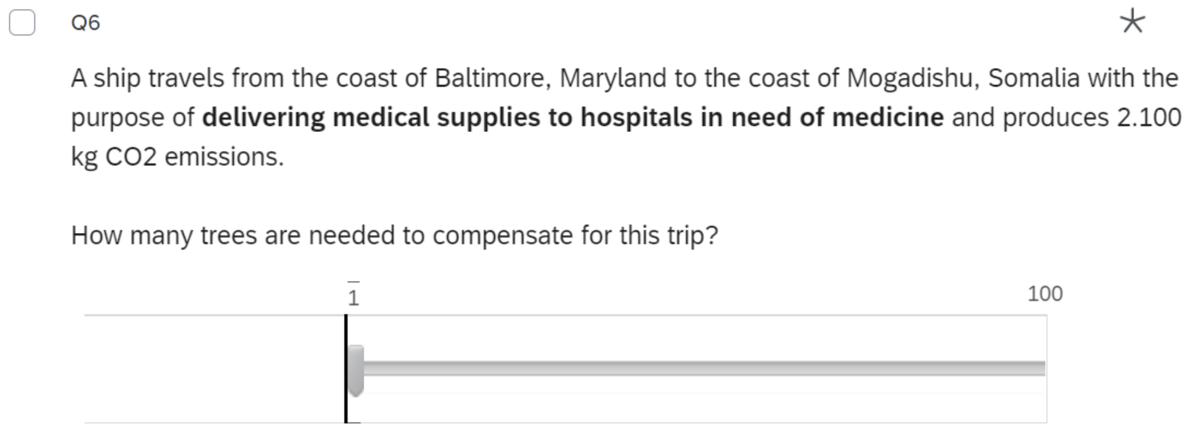
Q6 *

A ship travels from the coast of Baltimore, Maryland to the coast of Mogadishu, Somalia with the purpose of **delivering medical supplies to hospitals in need of medicine** and produces 2.100 kg CO2 emissions.

How many trees are needed to compensate for this trip?

1 100

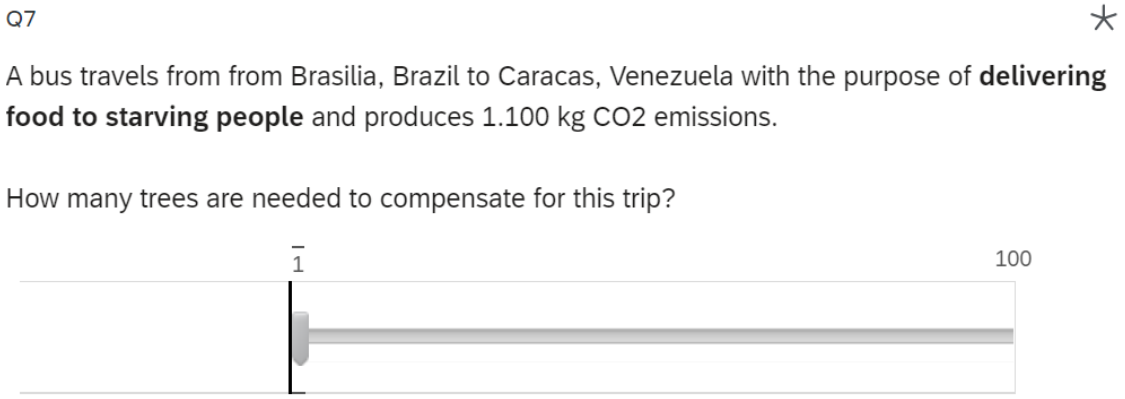
Q7 *

A bus travels from from Brasilia, Brazil to Caracas, Venezuela with the purpose of **delivering food to starving people** and produces 1.100 kg CO2 emissions.

How many trees are needed to compensate for this trip?

1 100

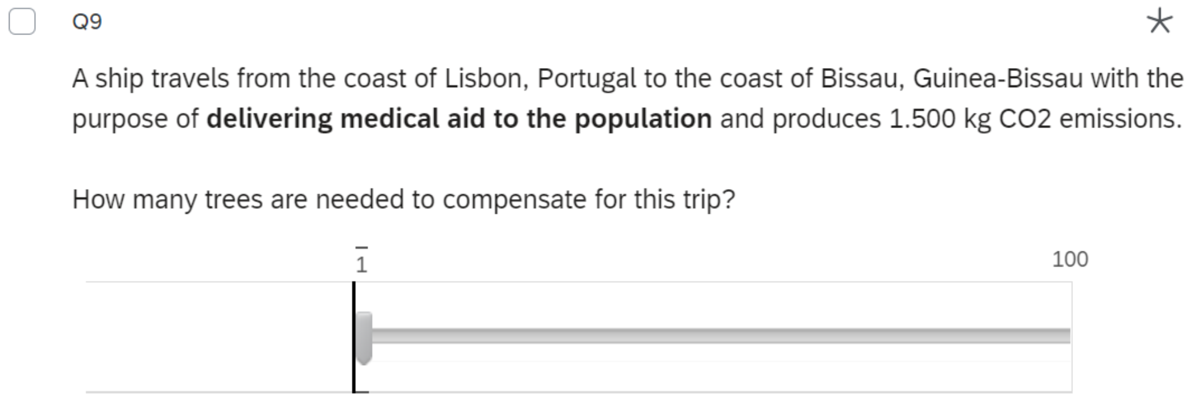
Q9 *

A ship travels from the coast of Lisbon, Portugal to the coast of Bissau, Guinea-Bissau with the purpose of **delivering medical aid to the population** and produces 1.500 kg CO2 emissions.

How many trees are needed to compensate for this trip?

1 100

Q3 *

An aeroplane travels from Paris, France to Aden, Yemen with the purpose of **delivering food to starving children** and produces 1.200 kg CO2 emissions.

How many trees are needed to compensate for this flight?

1 — 100

Q5 *

A bus travels from Austin, Texas to Los Pobladores, Mexico with the purpose **of building schools in a poor community** and produces 1000 kg CO2 emissions.

How many trees are needed to compensate for this trip?

1 — 100

Q8 *

An aeroplane travels from Toronto, Canada to Aleppo, Syria with the purpose of **re-building hospitals which were destroyed in the war** and produces 2.500 kg CO2 emissions.

How many trees are needed to compensate for this flight?

1 — 100

Below you can find both the objective carbon literacy measure and the self assessed knowledge and attitudes measure of carbon literacy derived from Sharp and Wheeler (2013):

Objective carbon literacy measure

Life cycle of household items

Out of the following products used around the home, which do you think emits the most Green House Gases, throughout their product lifecycle? RESPONSE SET
1. Washing machines, fridges, freezers and cooking;

2. Light bulbs, audio-visual equipment, etc;

3. Garden chemicals;

4. Carpets and rugs;

5. Furniture including wood, upholstered, mattress, metal, etc.;

6. Plastic products;

7. Forestry and wood products, such as partitions and wood;

8. Fabricated textile products, curtains and draperies.

Activities in the kitchen

In the average household kitchen, which act do you think emits the most Green House Gases? RESPONSE SET
(1) Cooking; (2) Chilling; (3) Dishwashing.

Life cycle of household activities

Which category do you think emits the most Green House Gases throughout its lifecycle? RESPONSE SET
1. Home Improvement;

2. Everyday home products such as washing machines, fridges,

showers, etc.;

3. Building new houses;

4. Groceries;

5. Clothing and footwear production.

Food life cycle stages

Thinking about the different lifecycle stages of all of the food and drink consumed, which lifecycle stage do you think

produces the most Green House Gases? RESPONSE SET
1. Raw materials (ingredients);

2. Use (chill, cook and wash energy);

3. Manufacturing;

4. Packaging;

5. Distribution;

6. End of life (disposal).

Grocery items

Now thinking specifically about the supermarket.....Out of the following grocery product categories, which do you think emits more Green House Gases when you consider it from being a raw material, through manufacturing and right to it being in the supermarket? RESPONSE SET
1. Dairy;

2. Meat and poultry;

3. Ready to eat food;

4. Bakery;

5. Alcoholic beverages;

6. Ready to eat fruit and vegetables;

7. Non-alcoholic beverages;

8. Fruit & veg that you usually cook;

9. Fish and seafood;

10. Other chilled and frozen foods

Animal products

Which of the following animal products emits the most Green House Gases per kilogram of product? RESPONSE SET
1. Beef;

2. Lamb;

3. Cheese;

4. Farmed salmon;

5. Pork;

6. Turkey;

7. Cnned tuna;

8. Chicken;

9. Eggs.

Self assessed knowledge and attitudes (on a scale from 1 – completely disagree to 9 – completely agree)

1. Amongst my friends, I am seen as a bit of an expert on environmental issues.
2. There's something I can do about the environment as an individual
3. It's worth me doing things to help the environment even if others don't do the same.
4. I believe there is a real link between the energy I use at home and climate change.
5. Effects of climate change are too far in the future to worry me.
6. I can feel a growing pressure to change the way I live to reduce the impact of climate change.
7. I don't find it hard to change my habits to be more environmentally friendly.
8. Government should take environmental concerns into account when making decisions
9. I am very concerned about the state of the environment at the moment.
10. I feel 'very well informed' about climate change
